# Novel gene re-arrangement in the mitochondrial genome of *Pisidiaserratifrons* (Anomura, Galatheoidea, Porcellanidae) and phylogenetic associations in Anomura

**DOI:** 10.3897/BDJ.11.e96231

**Published:** 2023-02-22

**Authors:** Jiayin lü, Xiangli Dong, Jiji Li, Yingying Ye, Kaida Xu

**Affiliations:** 1 Zhejiang Ocean University, Zhoushan, China Zhejiang Ocean University Zhoushan China

**Keywords:** Anomura, Galatheoidea, phylogenetic, gene rearrangement, divergence time analysis

## Abstract

To improve the taxonomy and systematics of Porcellanidae within the evolution of Anomura, we describe the complete mitochondrial genomes (mitogenomes) sequence of *Pisidiaserratifrons*, which is 15,344 bp in size, contains the entire set of 37 genes and has an AT-rich region. Compared with the pancrustacean ground pattern, at least five gene clusters (or genes) are significantly different with the typical genes, involving eleven tRNA genes and four PCGs and the tandem duplication/random loss and recombination models were used to explain the observed large-scale gene re-arrangements. The phylogenetic results showed that all Porcellanidae species clustered together as a group with well nodal support. Most Anomura superfamilies were found to be monophyletic, except Paguroidea. Divergence time estimation implies that the age of Anomura is over 225 MYA, dating back to at least the late Triassic. Most of the extant superfamilies and families arose during the late Cretaceous to early Tertiary. In general, the results obtained in this study will contribute to a better understanding of gene re-arrangements in Porcellanidae mitogenomes and provide new insights into the phylogeny of Anomura.

## Introduction

The infraorder Anomura is a highly diverse group of decapod crustaceans, including seven superfamilies, 20 families, 335 genera and more than 2500 species in total, some of the king crab and squat lobster being economically important (Dawson 1989, Lovrich 1997, Poore et al. 2011). However, the phylogenetic relationships within Anomura remain controversial. Earlier, based on adult morphological characteristics, classifications often differed in high-level composition (Baba 2008). Recently, more and more molecular and morphological data have been used to reconstruct the phylogeny of Anomura (Schnabel and Ahyong 2010, Kim et al. 2013, Gong et al. 2019). Although the monophyly of Anomura is widely accepted, phylogenetic relationships at high taxonomic levels remain unresolved, is dynamic and under continuous debate. Initially, the superfamily Galatheoidea was divided into seven families (i.e. Galatheidae, Munididae, Munidopsidae, Porcellanidae, Aeglidae, Chirostylidae and Kiwaidae) (Macpherson et al. 2005, Schnabel et al. 2011). Subsequently, Chirostylidae and Kiwaidae were removed to superfamily Chirostylidea, while Aeglidae was removed to Aegloidea (Pérez-Losada et al. 2002, McLaughlin et al. 2007). The current classification scheme divides Galatheoidea into Galatheidae (squat lobsters), Munididae, Munidopsidae and Porcellanidae (porcelain crabs) (Ahyong et al. 2010). So far, the phylogenetic relationship of some families in Anomura is still unclear. Therefore, data from sufficient species are required for a comprehensive phylogenetic analysis of the infraorder Anomura.

The porcelain crab (*Pisidiaserratifrons*) is one of the marine crustaceans that live in shallow waters less than 200 metres, with various habitats, which belong to the subphylum Crustacea, order Decapoda, infraorder Anomura, family Porcellanidae, genus Pisidia (Kim and Ko 2011). *P.serratifrons* is mainly distributed in the southern Korea, southern Japan and the southeast coastal region of China (Morton 1997, Qing et al. 2016). So far, most studies of this species have focused on morphology and growth (Morton 1997, Kim and Ko 2011).

The mitogenome of metazoans is usually 14–20 kb in size and encoded with a set of 37 genes, including 13 protein-coding genes (PCGs) (*cox1-3*, *cob*, *nad1-6*, *nad4L*, *atp6* and *atp8*), two ribosomal RNA genes (*rrns* and *rrnl*), 22 transport RNA genes (tRNAs) and an AT-rich region (also called D-loop region, CR) which contains some initiation sites for transcription and replication of the genome (Smith and Smith 2002, Sato and Sato 2013). Due to its haploid properties, matrilineal inheritance and rapid evolutionary rate, the mitogenome is increasingly being used in re-arrangement trends and phylogenetic analyses. With the rapid development of sequencing technology, more and more complete mitogenome sequences have been used in comparative genomics, molecular evolution and phylogeny (Tan et al. 2019).

Gene re-arrangements in the Anomura mitogenomes are relatively common (Arndt and Smith 1998, Hickerson and Cunningham 2000). As the sequence of animal mitogenomes remains stable over a long period of time and a complex shared derivative gene sequence is unlikely to appear independently in different pedigrees, gene re-arrangements can be used as an indicator to clarify the interspecific relationship. So far, several hypotheses have been suggested to help explain gene re-arrangements in animal mitogenomes. The recombination model and tandem duplication/random loss (TDRL) model are more commonly accepted. Recombination models are involved in the breaking and reconnecting of DNA strands. The TDRL model assumes that the re-arranged gene order occurs via tandem duplications followed by random deletion of certain duplications (Moritz and Brown 1987). This model has been widely used to explain the translocation of genes encoded on the same strand (Posada and Crandall 1998).

In this study, we successfully sequenced the complete mitogenome of *P.serratifrons*. In addition, the gene structure and gene re-arrangement of the mitogenome of *P.serratifrons* have been reported and a phylogenetic analysis of 31 Anomura species has been conducted, based on the nucleotide sequences of 13 PCGs. Based on the similarities and differences of the gene re-arrangement order in the mitogenome, the possible re-arrangement process was discussed in order to have a better understanding of the re-arrangement events and evolutionary mechanisms of the Anomura mitogenome.

## Materials and methods

### Sampling and DNA extraction

A specimen of *P.serratifrons* was collected from Zhoushan, Zhejiang Province, China (29°98′30N, 122°96′99″E). The specimen was immediately preserved in absolute ethanol after collection and then stored at −20°C. This specimen was identified by morphology and fresh tissues were dissected from the operculum and preserved in absolute ethanol before DNA extraction. The total genomic DNA was extracted using the salt-extraction procedure (Aljanabi and Martinez 1997) with a slight modification and stored at −20°C.

### Genome sequencing, assembly and annotation

The mitogenomes of *P.serratifrons* was sequenced by Origin gene Co. Ltd., Shanghai, China and was sequenced on the Illumina HiSeq X Ten platform. HiSeq X Ten libraries with an insert size of 300-500 bp were generated from the genomic DNA. About 10 Gb of raw data were generated for each library. Low-quality reads, adapters and sequences with high “N” ratios and length less than 25 bp were removed. The clean reads were assembled using the software NOVOPlasty (Dierckxsens et al. 2017) (https://github.com/ndierckx/NOVOPlasty) and annotated and manually corrected on the basis of the complete mitogenome sets, assembled de novo by using MITOS tools (Bernt et al. 2012) (MITOS Web Server (uni-leipzig.de)). To confirm the correct sequences, we compared the assembled mitochondrial genes with those of other Porcellanidae species and identified the mitogenomic sequences by checking the *cox1* barcode sequence with NCBI BLAST (Altschul et al. 1997). The abnormal start and stop codons were determined by comparing them with the start and stop codons of other marine crustacea. Then, the reads were reconstructed using the de novo assembly programme. The complete mtDNA was annotated using the software Sequin version 16.0. The mitogenome map of the *P.serratifrons* was drawn using the online tool Poksee (https://proksee.ca) (Grant and Stothard 2008). The secondary structures predicted of the tRNA genes were plotted by using MITOS Web Server. The relative synonymous codon usage (RSCU) values and Substitution saturation for the 13 PCGs were calculated by DAMBE 5 and analysed with MEGA 7 (Kumar et al. 2016). The GC-skews and AT-skews were used to determine the base compositional difference and strand asymmetry amongst the samples. According to the following formulae, Composition skew values were calculated: AT-skew = [A−T]/[A+T] and GC skew = [G−C]/[G+C]. Substitution saturation for the 13 PCGs was calculated by DAMBE 5 (Xia and Xie 2001).

### Phylogenetic analysis

The phylogenetic relationship within Anomura was reconstructed using the sequences of the 13 PCGs of a total of 34 complete mitogenome sequences downloaded from the GenBank database (https://www.ncbi.nlm.nih.gov/genbank/) and adding two species of Ocypodea to serve as the outgroup (Suppl. material 2). The phylogenetic relationships were analysed with Maximum Likelihood (ML) by using IQ-TREE 1.6.2 and Bayesian Inference (BI) methods in MrBayes 3.2 version programme (Perna and Kocher 1995, Huelsenbeck and Ronquist 2001, Nguyen et al. 2015). The ML analysis was inferred with 1000 ultrafast likelihood bootstrap replicates by using IQ-TREE 1.6.2. The best-fit model for each partition was K3Pu+f+R4, selected according to the Bayesian Information Criterion (BIC). BI was performed in MrBayes 3.2 and the best-fit evolutionary models were determined using MrMTgui (Ronquist et al. 2012). MrMTgui was used to associate PAUP, ModelTest and MrModelTest across platforms. MrBayes settings for the best-fit model (GTR + I + G) were selected by AIC in MrModelTest 2.3 (Nylander et al. 2004). The Bayesian phylogenetic analyses were performed using the parameter values estimated with the commands in MrModelTest or ModelTest (nst = 6, rates = invgamma) (Posada and Crandall 1998). Each with three hot chains and one cold chain were run simultaneously twice by Markov Chain Monte Carlo (MCMC) sampling and the posterior distribution was estimated. The MCMC chains were set for 2,000,000 generations and sampled every 1000 steps, with a relative burn-in of 25%. The convergence of the independent runs was evaluated by the mean standard deviation of the split frequencies (< 0.01). The phylogenetic trees were visualised and edited using Figure Tree v.1.4.3 software (Rambaut 2017).

### Divergence time estimation

The divergence times of Anomura were estimated with the programme BEAST v.1.10.4 (Joseph and Drummond 2011) under the uncorrelated strict clock model and fossil calibration points were used (Suppl. material 3), including with a normal prior distribution. The HKY substitution model was selected using based on BEAUti software and the Yule speciation process model. This study ran four independent Markov Chain Monte Carlo (MCMC) algorithms, the chain length of Markov Chain setting is 800,000,000 generations and sampled every 8000 generations. The first 10% of the trees were discarded as burn-in and each parameter was examined by the effective sample size (ESS) (> 200, as recommended) in Tracer v.1.6. Trees were assessed using TreeAnnotator and a chronogram was constructed in FigTree v.1.4.2.

## Results and discussion

### Genome structure and composition

**Figure 1.**
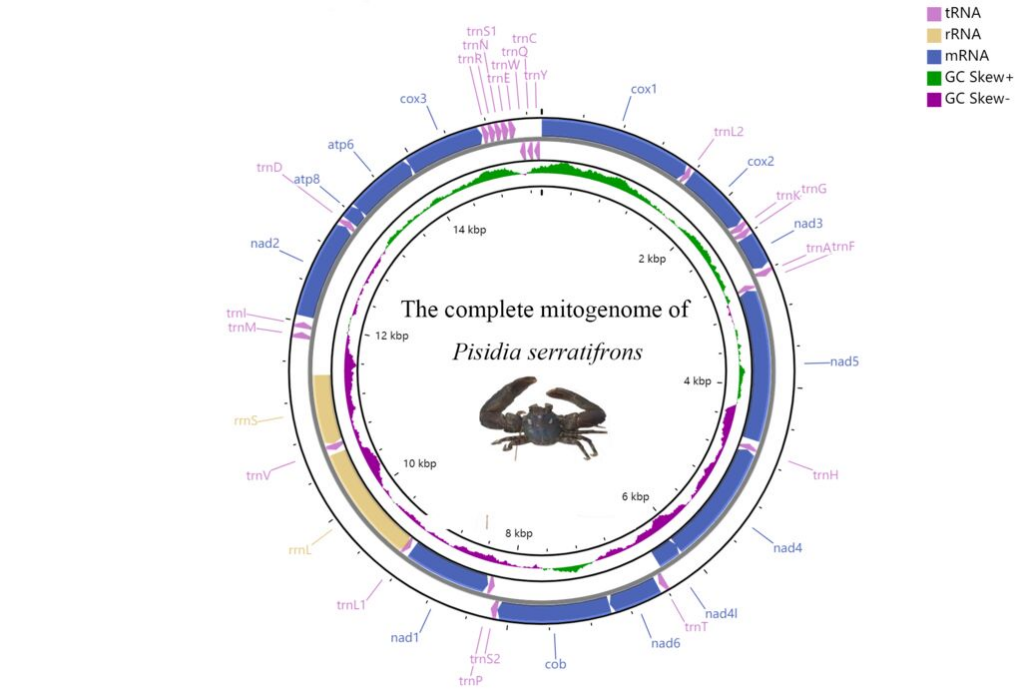
Circular mitogenome map of *P.serratifrons*. Protein coding, ribosomal and tRNA genes are shown with standard abbreviations. Arrows indicate the orientation of gene transcription. The inner circles show the G-C content and GC-skew, which are plotted as the deviation from the average value of the entire sequence.

The complete mitogenome sequence of *P.serratifrons* is a typical closed-circular molecule of 15，344 bp in size (GenBank accession number OM461359), which is a similar length to the published Porcellanidae mitogenomes (Tan et al. 2014, Lee et al. 2016), ranging from 15,324 to 15,348bp (Suppl. material 2). The mitogenome contents (Table 2) of *P.serratifrons* is the same as most published Anomura (Hickerson and Cunningham 2000, Yang et al. 2008, Kim et al. 2013), including 37 genes, 13PCGs, 22 tRNAs and two rRNA (*rrnl* and *rrns*), as well as a brief non-coding region. All the genes were identified and shown in Fig. 1 and Table 1. Most of the 37 genes are located on the heavy (H-) strand, except four PCGs (i.e. *nad5, nad4, nad4l* and *nad1*), eight tRNAs (i.e. *tRNA-Phe, His, Pro, Leu, Val, Gln, Cys* and *Tyr*) and two rRNA which are located on the light (L-) strand (Fig. 1). There are seven regions with overlap in the total *P.serratifrons* mitogenome, with one of them more than 10 bp *trnL1* (23 bp) and the other six shorter than 10 bp *nad4/atp8* (7 bp), *cox1* (5 bp), *cob* (2 bp) and *trnF/atp6* (1 bp) (Table 1). The *P.serratifrons* mitogenome also contains 376 bp of intergenic spacers located in 20 regions, ranging from 1 to 57 bp (Table 1) and indicating the occurrence of tandem duplications and the deletions of redundant genes. The nucleotide composition of the *P.serratifrons* mitogenome is A, 37.78%, T, 36.51%, G, 9.7% and C, 16.01%, with a high AT bias. The A + T (%) content of the mitogenomes was 74.29%. The AT-skew and GC-skew values are calculated for the chosen complete mitogenomes (Table 2). AT-skew of the *P.serratifrons* mitogenome is positive (0.017) and GC-skew of the *P.serratifrons* mitogenome is negative (−0.246), informing Ts and Cs are more abundant than Ts and Gs.

### PCGs and codon usage

**Figure 2.**
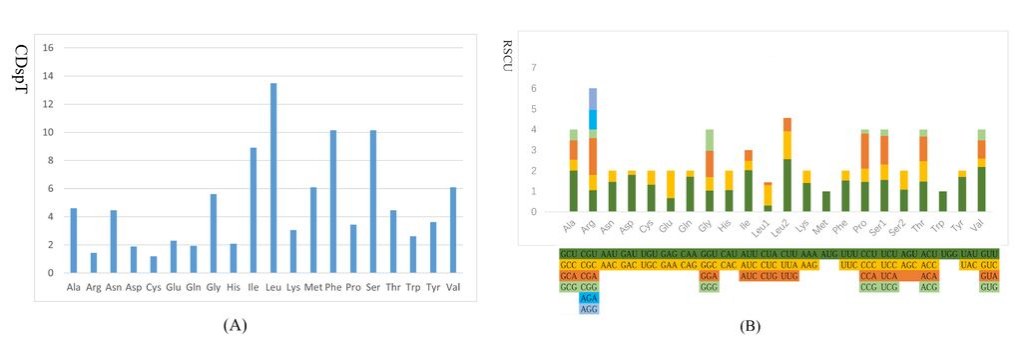
Codon usage patterns in the mitogenome of *P.serratifrons* CDspT, codons per thousand codons. Codon families are provided on the x-axis (**A**) and the relative synonymous codon usage (RSCU) (**B**).

The initial and terminal codons of all PCGs of *P.serratifrons* are listed in Table 3. *P.serratifrons* has 13 PCGs in the typical order found in Anomura species, containing seven NADH dehydrogenase *(nad1-nad6, nad4L*), three cytochrome c-oxidases (*cox1-cox3*), two ATPases (*atp6, atp8*) and cytochrome b (*cob*). The total length of the 13 PCGs is 11077 bp. The length of the 13 PCGs ranges from 159 to 1680 bp. The average A+T content is 72.7%, ranging from 67.84% (*cox1*) to 84.28% (*atp8*) (Table 1). The AT-skew and GC-skew are −0.182 and 0.011, respectively (Table 3). All of the PCGs are initiated by the start codon ATN (ATT, ATG, ATA and ATC), except *cox1* (ACG) and *cob* (TTG)，which is consistent with the mitogenome of most invertebrate species (Kong et al. 2009, Lee et al. 2016) . The majority of the PCGs are terminated with TAA, whereas *nad1* uses TAG as the stop codon (Table 3). The most frequently used amino acid in *P.serratifrons* is *Leu* and the least common anion acid is *Cys* (Fig. 2). The relative synonymous codon usage (RSCU) values for *P.serratifrons* of the 13 PCGs are shown in Table 3 and Fig. 2. The three most frequently detected codons are CUU (*Leu*), whereas GCA (*Gln*) is the least common codon. Based on CDspT and RSCU, comparative analyses showed that the codon usage pattern of *P.serratifrons* is conserved. The codon usage patterns of 13 PCGs are similar to those of other Porcellanidae species (Tan et al. 2014).

### Transfer RNAs, ribosomal RNAs

**Figure 3.**
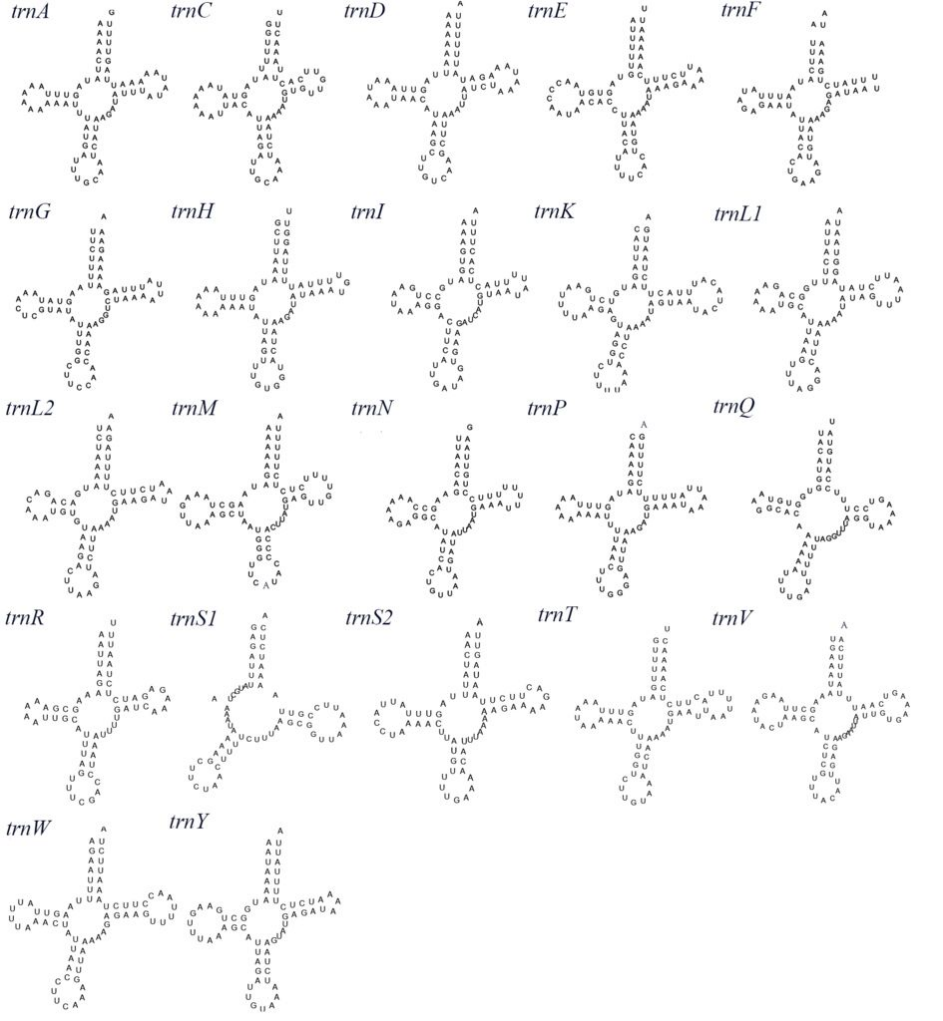
Putative secondary structures of tRNAs from the *P.serratifrons* mitogenome. The tRNAs are labelled with the abbreviations of their corresponding amino acids.

Like most Porcellanidae species, *P.serratifrons* mitogenome contains 22 tRNA genes (*Lee et al. 2016*). Fourteen of them are encoded by the heavy strain (H-) and the rest are encoded by the light strain (L-). In the whole mitogenome, the size of tRNAs ranges from 64 to 70 bp and has a total length of 1477 bp, with an obvious AT bias (76.98%) (Table 2). The AT-skew and GC-skew are 0.043 and 0.111, respectively, showing a slight bias towards the use of As and an apparent bias toward Gs (Table 2). The *trnS1* cannot form a secondary structure due to the lack of dihydrouracil (DHU) arms, while other tRNAs are capable of folding into a typical clover-leaf secondary structure (Fig. 3). The variation of *trnS1* structure is consistent with the *trnS1* structure reported in other invertebrate mitogenomes (Yang et al. 2008, Tan et al. 2019). The *rrns* and *rrnl* are 776 and 1303 bp, respectively, which are typically separated by *tRNA-Val* (Table 1). These sizes are similar to those of other Porcellanidae species. The A-T content of rRNAs is 77.73%. The AT-skew and GC-skew are 0.025 and 0.374, respectively, suggesting a slight bias towards the use of As and an apparent bias toward Gs (Table 2). As in most typical mitogenomes of other crabs, CR is located between *rrnS* and *tRNA-Met*. The 371 bp CR is obviously AT biased (77.63%). The AT-skew and GC-skew are −0.143 and −0.320, respectively, indicating an obvious bias towards the use of Ts and Cs. The index of substitution saturation (Iss) was measured as an implemention in DAMBE 5 and the GTR substitution model Iss is for the combined dataset of all PCGs of the 31 Anomura mitogenomes and was signifcantly lower (Iss = 0.647) than the critical values (Iss, cSym = 0.879). The genes are not saturated, so the reconstructed phylogeny was reliable.

### Gene re-arrangement

**Figure 4.**
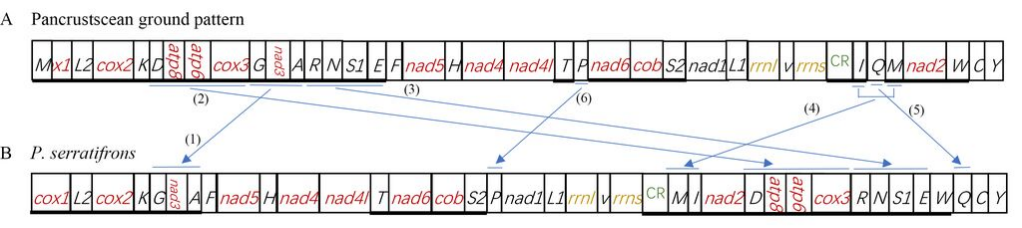
Gene re-arrangements in *P.serratifrons* mitogenome. Gene re-arrangement steps: **A** ancestral gene arrangement of crustaceans; **B** gene order in the *P.serratifrons* mitogenome.

**Figure 5.**
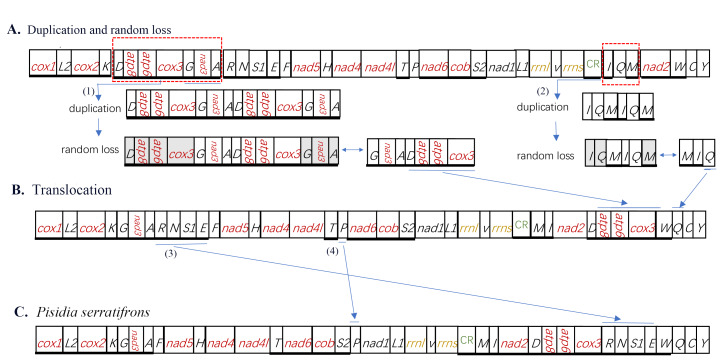
Inferred intermediate steps between the ancestral gene arrangement of crustaceans and *P.serratifrons* mitogenome. **A** Duplication-loss and translocation in the ancestral mitogenome of crustaceans. The duplicated gene block is boxed in dash and the lost genes are labelled with grey **B** Translocation; **C** The final gene order in the *P.serratifrons* mitogenome.

Compared with the gene arrangement in the ancestral crustaceans (pancrustacean ground pattern), we found that the gene order in *P.serratifrons* mitogenome underwent a massive re-arrangement. As Fig. 4 shows, at least five gene clusters (or genes) are significantly different from the typical genes, involving eleven tRNA genes (*D*, *G, A, R, N, S1, E, P, I, Q* and *M*) and four PCGs (*atp8, atp6, cox3* and *nad3*) (Fig. 4). The re-arrangement of the five gene clusters (or genes) is as follows (Fig. 5): (1) The *G-nad3-A* gene cluster moved to downstream of *K*; (2) The *D-atp8-atp6-cox3* gene cluster shift to downstream of *nad2*; (3) Four tRNA clusters (*R-N-S1-E*) shifted upstream of *W*; (4) The *I-Q-M* cluster was divided into two sections, the *I-Q-M* cluster order was changed into *M-I-Q* and then a single *Q* was moved to downstream of *W*; (5) A single *P* moved from the downstream of *T* to downstream of the *S_2_*.

At present, there are three models to explain the mitochondrial genome re-arrangement: (1) replication-random loss model (Moritz and Brown 1987); (2) duplication-non-random loss (Lavrov et al. 2002); (3) recombination (Rokas et al. 2003). Based on the mitochondrial sequence characteristics of *P.serratifrons*, we concluded that replication-random loss and recombination resulted in the generation of the re-arrangement phenomenon. Firstly, two gene clusters underwent a complete copy, forming two dimeric blocks, (*D-atp8-atp6-cox3-G-nad3-A*) - (*D-atp8-atp6-cox3-G-nad3- A*) and (*I-Q-M*) - (*I-Q-M*) (Fig. 5). Due to the parsimony of the mitochondrial genome, usually only one gene is active, while the other gene has lost its original function and evolution in the genome random loss of genes can occur along the way. This process can be shown as *D-atp8-atp6-cox3-G-nad3-A-D-atp8-atp6-cox3-G-nad3-A, I-Q-M-I-Q-M* (underline denotes the deleted gene) with formation of two new gene blocks (*G-nad3-A-D-atp8-atp6-cox3*) and (*M-I-Q*). Tandem duplication followed by random loss is widely used to explain this type of translocation of mitochondrial genes (Kong et al. 2009, Shi et al. 2015, Sun et al. 2019). Therefore, we ascertain that the duplication-random loss model is the most likely explanation for these two gene block re-arrangements. Then, the two new gene blocks result in a translocation. (*G-nad3-A-D-atp8-atp6-cox3*) block is translocated downstream to the *nad2*, leaving *G-nad3-A* in the original position. (*M-I-Q*) block is translocated to upstream of *W*, leaving *M-I* in the original position. In the second step, four tRNA clusters (*R-N-S_1_-E*) shifted to upstream of *W. P* is translocated to downstream of *S_2_*. Finally, the ultimate gene arrangement of the *P.serratifrons* mitogenome is shown in Fig. 5C.

**Figure 6.**
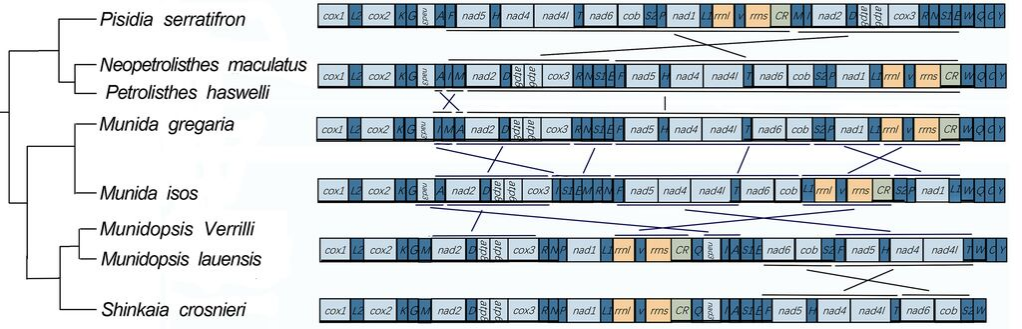
Mitochondrial gene arrangements of eight species in Galatheoidea. Gene arrangement of all genes are transcribed from left to right. The re-arranged gene blocks are underlined and compared with ancestral gene arrangement of Anomura.

Comparing mitochondrial gene order has been proved to be a valuable tool in crustacean phylogeny. Based on the comparative analysis of mitochondrial gene arrangement within Galatheoidea, we found that eight Galatheoidea mitogenomes showed a massive re-arrangement, which differs from any gene order ever reported in decapods (Fig. 6). Amongst the eight gene re-arrangement patterns in this study, the *(F-nad5-H-nad4-nad4L*) and (*rrnL-V-rrnS*) regions are extremely conserved, which is consistent with the conclusion of Shao et al. (2001) that the (*F-nad5-H-nad4-nad4L*) and (*rrnL-V-rrnS*) regions are considered extremely conserved in animals. The *P.serratifrons* mitochondrial gene arrangement is closest to *Neopetrolisthesmaculatus* and *Petrolistheshaswelli* which provides further support for the close relationship. The mitochondrial gene orders of *Munidagregaria* shared the most similarities with *Munidaisos*, while *Munidopsis Verrilli* and *Munidopsislauensis* shared higher similarities with *Shinkaiacrosnieri*. These results are consistent with the conclusion from the gene order based phylogenetic tree. The gene order of the Munididae has a complex within-genus re-arrangement which seems to be related to their particular habitat. Our results support the fact that those comparisons of mitochondrial gene re-arrangements are a useful tool for phylogenetic studies.

### Phylogenetic relationships

**Figure 7.**
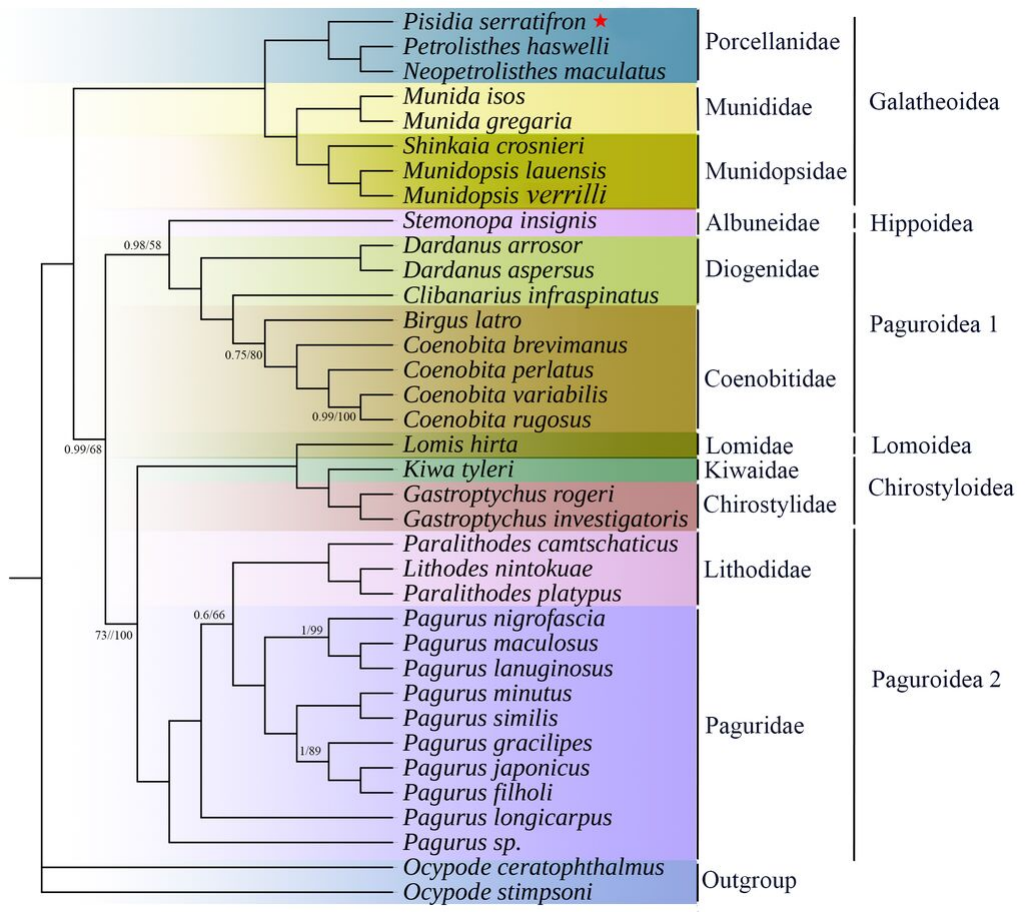
The phylogenetic tree was inferred from the nucleotide sequences of 13 mitogenome PCGs using BI and ML methods. Numbers on branches indicate posterior probability (BI) and bootstrap support (ML).

In the present study, the phylogenetic relationships were analysed, based on the sequences of the 13 PCGs to clarify the relationships in Anomura. *P.serratifrons* and another 31 known Anomura species were analysed, with *O.ceratophthalmus* and *Q.stimpsoni* as outgroups. The two phylogenetic trees (i.e. Maximum Likelihood (ML) tree and Bayesian Inference (BI) tree) resulted in identical topological structuring with different supporting value. Subsequently, only one topology (ML) with both support values was presented and displayed (Fig. 7). It was obvious that *P.serratifronsa*, *N.maculatus* and *P.haswelli* formed a Porcellanidae clade with high support value. The families Munididae and Munidopsidae were grouped into one clade and Porcellanidaeas as the basal group which was similar to what was reported by McLaughlin et al. based on morphological characters and by Gong et al. based on the amino acid dataset of 13 PCGs (McLaughlin et al. 2007, Gong et al. 2019).

Amongst the 11 families included in our phylogenetic tree, each family in the tree forms a monophyletic clade with high nodal support values, except Paguridae. At a higher level of classification, most superfamilies from Anomura were found to be monophyletic, except Paguroidea, which is in line with previous studies (McLaughlin 1983, Tan et al. 2018). It showed that Paguroidea was divided into two clades, ((Coenobitidae + Diogenidae) + (Lithodidae + Paguridae)), which is consistent with previous results (Tan et al. 2018, Gong et al. 2019), while Tan et al. (2019) deem that Lithodidae was excluded from Paguroidea and belonged to a new superfamily Lithodoidea. Besides, our phylogenetic tree showed that (Porcellanidae + (Munidopsidae + Munididae)) formed a Galatheoidea clade in this tree and (Chirostylidae+ Kiwaidae) formed Chirostylidea in a clade which was consistent with Sun et al. (2019) (based on morphological characters) and Schnabel et al. (2011) (based on mitochondrial 16S rRNA and nuclear 18S and 28S rRNA), while the monophyly of Galatheoidea is still not recognised by some studies, mainly due to the classification of Chirostylidae. According Tan et al. (2018), they regarded Chirostylidae as a member of the Galatheoidea and Galatheoidea formed a polyphyletic clade in their studies.

### Divergence time estimation

**Figure 8.**
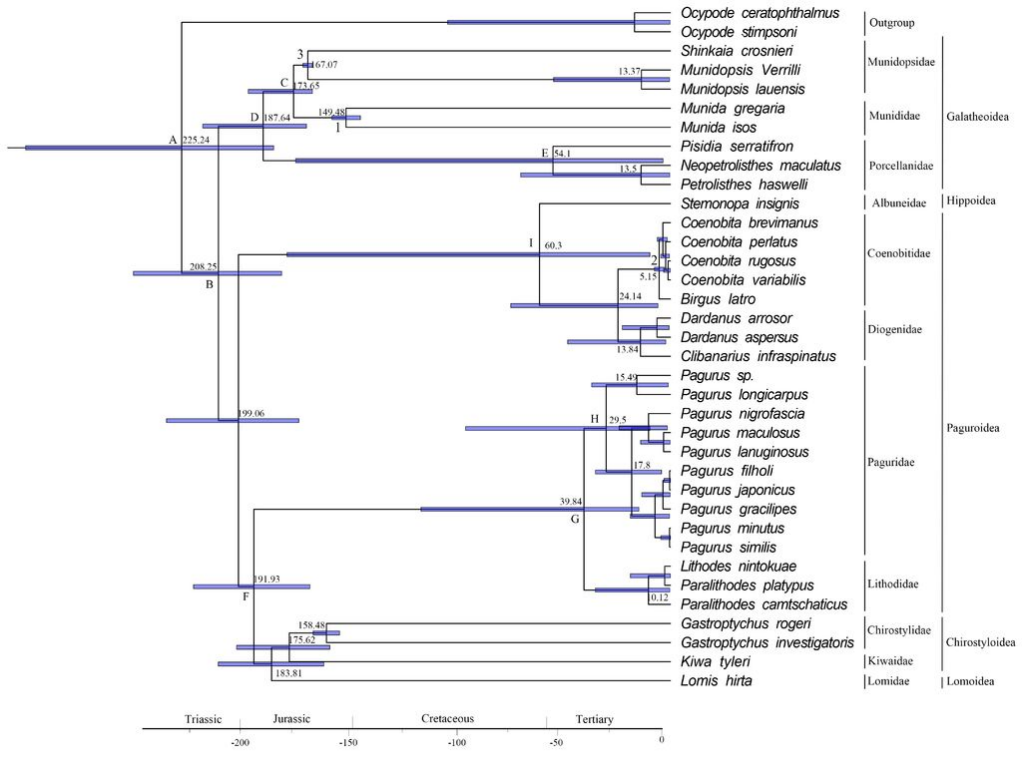
Anomura divergence time estimated using the Bayesian relaxed-molecular clock method. The 95% confidence intervals for each node are shown in light blue bars. 1-3: 3 fossil calibration nodes (Corresponding to Suppl. material 3).

The divergence time analysis, based on 13 PCGs of the mitochondrial genome, implies that the divergence of Anomura occurred in the early Triassic (~ 225.2 MYA, 95% credibility interval = 182.79–297.16 MYA, Fig. 8A), which is roughly the same as the conclusion of Bracken-Grissom et al. (2013) that the origin of Anomura is Late Permian ~ 259 (224-296) MYA, based on the divergence time analysis. The Galatheoidea superfamily diverged in the early Jurassic (208 Ma, 95% credibility interval = 167.73-215.52 MYA, Fig. 8B), into the Munidopsidae and Munididae during the Early Jurassic (~ 173 MYA, Fig. 8C), while the family Procellanidae diverged in the Early Jurassic (~ 187 MYA, Fig. 8D) with rapid speciation of present day species occurring since the mid-Miocene (~ 54 MYA, Fig. 8E). The Lomidae, Kiwaidae and Chirostylidae all originated in the Jurassic (~ 183.81 MYA, 175.62 Ma and 158.48 Ma, respectively). The hermit crab formed two subclades during the Jurassic period (~ 191 MYA, Fig. 8 F), the first subclade branches being composed of Lithodidae and Paguridae. The most recent common ancestor of Lithodidae and Paguridae was divided into a new family in the Middle Tertiary (~ 39.84 MYA, Fig. 8G). The Paguridae was first discovered in the Tertiary (~ 29.5 MYA, Fig. 8H). The second subclade was formed by the hermit crabs in the middle Cretaceous (~ 60.3 MYA, Fig. 8I) and differentiation formed the family of Albuneidae, Coenobitidae and Diogenidae. The differentiation time was longer than that of the first subclade and appeared about 20 MYA earlier. The results support the multi-family origin of the hermit crab.

## Conclusion

In this study, the mitogenome of *P.serratifrons* was sequenced by next-generation sequencing, thereby generating new mitochondrial data for Porcellanidae. We analysed the mitogenome of *P.serratifrons* and found it is similar to other Anomura with many significant features including AT-skew, a codon usage bias etc. Compared with the pancrustacean ground pattern, the gene order in *P.serratifrons* mitogenome underwent a massive re-arrangement. The Galatheoidea showed eight re-arrangement patterns and their re-arrangement similarity is consistent with phylogenetic relationships. Our phylogenetic tree had similarities and disagreements with predecessor studies. The phylogenetic analyses indicated that *P.serratifronsa*, *N.maculatus* and *P.haswelli* formed a Porcellanidae clade. Divergence time estimation implies that the age of Anomura is over 225 MYA, dating back to at least the late Triassic. Most of the extant superfamilies and families arose during the late Cretaceous to early Tertiary. These results provide insight into the gene arrangement features of Anomura mitogenomes and lay the foundation for further phylogenetic studies on Anomura.

## Data availability

Suppl. material 1. The complete mitogenome of *Pisidiaserratifrons* has been submitted to GenBank under the accession number of OM461359.

## Supplementary Material

3140D191-1A6D-5C14-A6F6-3202280E3B72 10.3897/BDJ.11.e96231.suppl1 Supplementary material 1 Mitogenome of *Pisidiaserratifrons* Data type genomic Brief description The complete mitogenome of *Pisidiaserratifrons* has been submitted to GenBank under the accession number of OM461359 File: oo_756076.fas https://binary.pensoft.net/file/756076 Jiayin LYu

9B11FD9A-9504-5157-AD62-549CD33DC221 10.3897/BDJ.11.e96231.suppl2 Supplementary material 2 List of 34 species and two outgroups used in this paper Data type table File: oo_777016.doc https://binary.pensoft.net/file/777016 Jiayin LYu

88AA600B-E454-5ECB-86C1-E2CD341690E7 10.3897/BDJ.11.e96231.suppl3 Supplementary material 3 Basic information on three fossil correction points Data type table File: oo_777017.docx https://binary.pensoft.net/file/777017 Jiayin LYu

## Figures and Tables

**Table 1.** Features of the mitochondrial genome of *P.serratifrons*.

Gene	Position		length	Amino acid	Start/stop codon	anticodon	Intergenic region	strand
	from	to						
cox1	1	1533	1533	510	ACG/TAA		-5	H
trnL2	1529	1592	64			TAA	3	H
cox2	1596	2280	685	228	ATG/T(AA)		0	H
trnK	2281	2351	71			TTT	3	H
trnG	2355	2421	67			TCC	0	H
nad3	2422	2772	351	116	ATT/TAA		23	H
trnA	2796	2862	67			TGC	3	H
trnF	2866	2929	64			GAA	-1	L
nad5	2929	4641	1713	570	ATG/TAA		18	L
trnH	4660	4725	66			GTG	2	L
nad4	4728	6068	1341	446	ATG/TAA		-7	L
nad4l	6062	6343	282	93	ATT/TAA		31	L
trnT	6375	6443	69			TGT	45	H
nad6	6489	6980	492	163	ATT/TAA		5	H
cob	6986	8122	1137	378	TTG/TAA		-2	H
trnS2	8121	8190	70			TGA	7	H
trnP	8198	8264	67			TGG	8	L
nad1	8273	9202	930	309	ATA/TAG		30	L
trnL1	9233	9298	66			TAG	-23	L
rrnL	9276	10578	1303				33	L
trnV	10612	10685	74			TAC	1	L
rrnS	10687	11462	776				0	L
CR	11463	11834	371				0	H
trnM	11834	11901	68			CAT	37	H
trnI	11939	12002	64			GAT	57	H
nad2	12060	13055	996	331	ATT/TAA		0	H
trnD	13056	13122	67			GAT	0	H
atp8	13123	13281	159	52	ATG/TAA		-7	H
atp6	13275	13949	675	224	ATG/TAA		-1	H
cox3	13949	14740	792	263	ATG/TAA		5	H
trnR	14746	14809	64			TCG	0	H
trnN	14810	14875	66			GTT	0	H
trnS1	14876	14940	65			TCT	0	H
trnE	14941	15010	70			TTC	3	H
trnW	15014	15083	70			TCA	15	H
trnQ	15099	15165	67			TTG	26	L
trnC	15182	15245	64			GCT	12	L
trnY	15258	15324	67			GTA	0	L

**Table 2.** Composition and skewness of *P.serratifrons* mitogenome.

	A%	T%	G%	C%	(AT)%	AT-skew	GC-skew	Length (bp)
Mitogenome	37.78	36.51	9.7	16.01	74.29	0.017	-0.246	15344
PCGs	29.72	42.98	13.79	13.51	72.70	-0.182	0.011	11077
cox1	29.29	38.55	15.46	16.70	67.84	-0.137	-0.039	1533
cox2	34.26	36.35	12.12	17.37	70.51	-0.031	-0.178	685
atp8	41.51	42.77	6.92	8.81	84.28	-0.015	-0.120	159
atp6	30.96	41.63	11.26	16.15	72.59	-0.147	-0.178	675
cox3	31.19	38.26	13.51	17.05	69.44	-0.102	-0.116	792
nad3	31.34	45.3	10.26	13.11	76.64	-0.280	0.341	351
nad5	28.51	46.90	15.60	8.99	75.42	-0.244	0.269	1680
nad4	26.10	48.32	17.30	8.28	74.42	-0.299	0.353	1341
nad4L	25.89	49.29	19.15	5.67	75.18	-0.311	0.543	282
nad6	31.98	44.19	7.17	16.67	76.16	-0.160	-0.398	516
cob	31.22	37.55	12.40	18.82	68.78	-0.092	-0.206	1137
nad1	26.02	46.24	18.60	9.14	72.26	-0.280	0.341	930
nad2	31.43	45.18	7.93	15.46	76.61	-0.180	-0.322	996
tRNAs	40.15	36.83	12.80	10.22	76.98	0.025	0.374	1477
rRNAs	39.83	37.90	15.30	6.97	77.73	-0.182	0.011	2079
AT-rich	31.62	42.16	8.92	17.30	77.78	-0.143	-0.320	371

**Table 3.** The codon number and relative synonymous codon usage in the mitochondrial genome of *P.serratifrons*.

**codon**	**count**	**RSCU**	**codon**	**count**	**RSCU**	**codon**	**count**	**RSCU**	**codon**	**count**	**RSCU**
UUU(F)	407	1.527	UCU(S)	98	1.549	UAU(Y)	224	1.697	GAA(E)	28	1.333
UUC(F)	126	0.473	UCC(S)	47	0.743	UAC(Y)	40	0.303	UGU(C)	73	1.315
CUA(L)	9	0.308	UCA(S)	88	1.391	UGA(*)	72	1.049	UGC(C)	38	0.685
CUC(L)	29	0.991	UCG(S)	20	0.316	UAG(*)	40	0.583	UGG(W)	63	1
CUG(L)	4	0.137	CCU(P)	30	1.463	UAA(*)	94	1.369	CGU(R)	10	1.053
CUU(L)	75	2.564	CCC(P)	13	0.634	CAU(H)	29	1.055	CGC(R)	7	0.737
UUA(L)	31	1.344	CCA(P)	35	1.707	CAC(H)	26	0.945	CGA(R)	17	1.789
UUG(L)	64	0.656	CCG(P)	4	0.195	CAA(Q)	29	1.706	CGG(R)	4	0.421
AUU(I)	255	2.029	ACU(T)	55	1.467	CAG(Q)	5	0.294	AGA(R)	58	0.967
AUC(I)	57	0.454	ACC(T)	37	0.987	AAU(N)	216	1.459	AGG®	62	1.033
AUA(I)	65	0.517	ACA(T)	45	1.2	AAC(N)	80	0.541	AGU(S)	84	1.084
AUG(M)	41	1	ACG(T)	13	0.347	AAA(K)	134	1.403	AGC(S)	71	0.916
GUU(V)	71	2.185	GCU(A)	34	2	AAG(K)	57	0.597	GGU(G)	29	1.036
GUC(V)	13	0.4	GCC(A)	9	0.529	GAU(D)	53	1.797	GGC(G)	18	0.643
GUA(V)	29	0.892	GCA(A)	16	0.941	GAC(D)	6	0.203	GGA(G)	36	1.286
GUG(V)	17	0.523	GCG(A)	9	0.529	GAG(E)	14	0.667	GGG(G)	29	1.036

## References

[B8151390] Ahyong Shane T., Baba Keiji, Macpherson Enrique, Poore Gary C. B. (2010). A new classification of the Galatheoidea (Crustacea: Decapoda: Anomura). Zootaxa.

[B8141926] Aljanabi Salah M., Martinez Iciar (1997). Universal and rapid salt-extraction of high quality genomic DNA for PCR-based techniques.. Nucleic Acids Research.

[B8141935] Altschul Stephen F., Madden Thomas L., Schäffer Alejandro A., Zhang Jinghui, Zhang Zheng, Miller Webb, Lipman David J. (1997). Gapped BLAST and PSI-BLAST: a new generation of protein database search programs.. Nucleic Acids Research.

[B8141947] Arndt A, Smith M J (1998). Mitochondrial gene rearrangement in the sea cucumber genus *Cucumaria*.. Molecular Biology and Evolution.

[B8142287] Baba Keiji (2008). Torbenella, a replacement name for *Torbenia* Baba, 2005 (Decapoda, Galatheidae) preoccupied by *Torbenia* Libert, 2000 (Insecta, Lepidoptera, Lycaenidae). Crustaceana.

[B8141956] Bernt Matthias, Donath Alexander, Jühling Frank, Externbrink Fabian, Florentz Catherine, Fritzsch Guido, Pütz Joern, Middendorf Martin, Stadler Peter F. (2012). MITOS: improved *de novo* metazoan mitochondrial genome annotation.. Molecular Phylogenetics and Rvolution.

[B8304753] Bracken-Grissom Heather D, Cannon Maren E, Cabezas Patricia (2013). A comprehensive and integrative reconstruction of evolutionary history for Anomura (Crustacea: Decapoda). Palaeontologische Zeitschrift.

[B8196099] Dawson Elliot W. (1989). King crabs of the world (Crustacea, Lithodidae) and their fisheries: A comprehensive bibliography.

[B8141979] Dierckxsens Nicolas, Mardulyn Patrick, Smits Guillaume (2017). NOVOPlasty: *de novo* assembly of organelle genomes from whole genome data.. Nucleic Acids Research.

[B8141988] Gong Li, Lu Xinting, Wang Zhifu, Zhu Kehua, Liu Liqin, Jiang Lihua, Lü Zhenming, Liu Bingjian (2019). Novel gene rearrangement in the mitochondrial genome of *Coenobitabrevimanus* (Anomura: Coenobitidae) and phylogenetic implications for Anomura.. Genomics.

[B8142001] Grant Jason R., Stothard Paul (2008). The CGView Server: a comparative genomics tool for circular genomes.. Nucleic Acids Research.

[B8142010] Hickerson M J, Cunningham C W (2000). Dramatic mitochondrial gene rearrangements in the hermit crab *Paguruslongicarpus* (Crustacea, Anomura).. Molecular Biology and Evolution.

[B8142019] Huelsenbeck J P, Ronquist F (2001). MRBAYES: Bayesian inference of phylogenetic trees.. Bioinformatics.

[B8169984] Joseph Heled, Drummond Alexei (2011). Calibrated tree priors for relaxed phylogenetics and divergence time estimation. Systematic Biology.

[B8151460] Kim Han-Ju, Ko Hyun-Sook (2011). Zoeal Stages of *Pisidiaserratifrons* (Crustacea: Decapoda: Porcellanidae) under Laboratory Conditions. Animal Systematics, Evolution and Diversity,.

[B8151442] Kim Sanghee, Choi Han-Gu, Park Joong-Ki, Min Gi-Sik (2013). The complete mitochondrial genome of the subarctic red king crab, *Paralithodescamtschaticus* (Decapoda, Anomura).. Mitochondrial DNA.

[B8142346] Kong Xiaoyu, Dong Xiaoli, Zhang Yanchun, Shi Wei, Wang Zhongming, Yu Ziniu (2009). A novel rearrangement in the mitochondrial genome of tongue sole, *Cynoglossussemilaevis*: control region translocation and a tRNA gene inversion. Genome.

[B8142037] Kumar Sudhir, Stecher Glen, Tamura Koichiro (2016). MEGA7: Molecular evolutionary genetics analysis version 7.0 for bigger datasets.. Molecular Biology and Evolution.

[B8142056] Lavrov Dennis V, Boore Jeffrey L, Brown Wesley M (2002). Complete mtDNA sequences of two millipedes suggest a new model for mitochondrial gene rearrangements: duplication and nonrandom loss.. Molecular Biology and Evolution.

[B8142047] Lee Chi Woo, Song Ji-Hun, Min Gi-Sik, Kim Sanghee (2016). The complete mitochondrial genome of squat lobster, *Munidagregaria* (Anomura, Galatheoidea, Munididae).. Mitochondrial DNA. Part B, Resources.

[B8142357] Lovrich Gustavo A. (1997). The mixed fishery for the king crabs *Lithodessantolla* and *Paralomisgranulosa* (Anomura: Lithodidae) in Tierra del Fuego, Argentina. Investigaciones Marinas.

[B8151798] Macpherson Enrique, Jones William, Segonzac Michel (2005). A new squat lobster family of Galatheoidea (Crustacea, Decapoda: Anomura) from the hydrothermal vents of the Pacific-Antarctic Ridge. Zoosystema.

[B8142409] McLaughlin Patsy A. (1983). Hermit crabs―are they really polyphyletic?. Journal of Crustacean Biology.

[B8142400] McLaughlin Patsy A, Lemaitre Rafael, Sorhannus Ulf (2007). Hermit crab phylogeny: a reappraisal and its “fall-out”. Journal of Crustacean Biology.

[B8142065] Moritz C, Brown W M (1987). Tandem duplications in animal mitochondrial DNAs: variation in incidence and gene content among lizards.. Proceedings of the National Academy of Sciences of the United States of America.

[B8142324] Morton Brian (1997). The marine flora and fauna of Hong Kong and Southern China.

[B8142418] Nguyen Lam-Tung, Schmidt Heiko A, Von Haeseler Arndt, Minh Bui Quang J (2015). IQ-TREE: a fast and effective stochastic algorithm for estimating maximum-likelihood phylogenies. Molecular Biology and Evolution.

[B8142437] Nylander Johan A. A., Ronquist Fredrik, Huelsenbeck John P, Nieves-Aldrey Joséluis (2004). Bayesian phylogenetic analysis of combined data. Systematic Biology.

[B8151451] Pérez-Losada Marcos, Jara Carlos G., Bond-Buckup Georgina, Porter Megan L. (2002). Phylogenetic position of the freshwater anomuran family Aeglidae. Journal of Crustacean Biology.

[B8142107] Perna N T, Kocher T D (1995). Patterns of nucleotide composition at fourfold degenerate sites of animal mitochondrial genomes.. Journal of Molecular Evolution.

[B8142173] Poore Gary C. B., Ahyong Shane T., Taylor Joanne Elizabeth (2011). The biology of squat lobsters. Csiro Publishing.

[B8142116] Posada D, Crandall K A (1998). MODELTEST: testing the model of DNA substitution.. Bioinformatics.

[B8288488] Qing Wu, Z Li, F Dai (2016). Taxonomic diversity of crustaceans in Yellow Sea and Bohai Sea. Biodiversity Science.

[B8142455] Rambaut Andrew (2017). FigTree-version 1.4. 3, a graphical viewer of phylogenetic trees. Computer program distributed by the author.

[B8142446] Rokas Antonis, Ladoukakis Emmanuel, Zouros Eleftherios (2003). Animal mitochondrial DNA recombination revisited. Trends in Ecology Evolution.

[B8151768] Ronquist Fredrik, Teslenko Maxim, Ayres Daniel L., A Marc, Darling Aaron, Darling Aaron (2012). MrBayes 3.2: efcient Bayesian phylogenetic inference and model choice across a large model space. Systematic Biology.

[B8142182] Sato Miyuki, Sato Ken (2013). Maternal inheritance of mitochondrial DNA by diverse mechanisms to eliminate paternal mitochondrial DNA.. Biochimica et Biophysica Acta.

[B8142468] Schnabel Kareen E., Ahyong Shane T., Maas Elizabeth W. (2011). Galatheoidea are not monophyletic–molecular and morphological phylogeny of the squat lobsters (Decapoda: Anomura) with recognition of a new superfamily. Molecular Phylogenetics Evolution.

[B8142477] Schnabel Kareen E, Ahyong Shane T (2010). A new classification of the Chirostyloidea (Crustacea: Decapoda: Anomura). Zootaxa.

[B8304692] Shao Renfu, Campbell Nick J. H., Schmidt Evan R. (2001). Increased rate of gene rearrangement in the mitochondrial genomes of three orders of hemipteroid insects. Molecular Biology & Evolution.

[B8142202] Shi Wei, Gong Li, Wang Shu-Ying, Miao Xian-Guang, Kong Xiao-Yu (2015). Tandem duplication and random loss for mitogenome rearrangement in Symphurus (Teleost: Pleuronectiformes).. BMC Genomics.

[B8142391] Smith J. Maynard, Smith N. H. (2002). Recombination in animal mitochondrial DNA. Molecular Biology and Evolution.

[B8142486] Sun Shao'e, Sha Zhongli, Wang Yanrong (2019). The complete mitochondrial genomes of two vent squat lobsters, *Munidopsislauensis* and *M.verrill*i: Novel gene arrangements and phylogenetic implications. Ecology and Evolution.

[B8142237] Tan Mun Hua, Gan Han Ming, Lee Yin Peng, Austin Christopher M (2014). The complete mitogenome of the porcelain crab *Petrolistheshaswelli* Miers, 1884 (Crustacea: Decapoda: Anomura).. Mitochondrial DNA. Part A, DNA Mapping, Sequencing, and Analysis.

[B8142212] Tan Mun Hua, Gan Han Ming, Lee Yin Peng, Linton Stuart, Grandjean Frederic, Bartholomei-Santos Marlise Ladvocat, Miller Adam D, Austin Christopher M (2018). ORDER within the chaos: Insights into phylogenetic relationships within the Anomura (Crustacea: Decapoda) from mitochondrial sequences and gene order rearrangements.. Molecular Phylogenetics and Evolution.

[B8142225] Tan Mun Hua, Gan Han Ming, Lee Yin Peng, Bracken-Grissom Heather, Chan Tin-Yam, Miller Adam D., Austin Christopher M. (2019). Comparative mitogenomics of the Decapoda reveals evolutionary heterogeneity in architecture and composition.. Scientific Reports.

[B8151759] Xia X., Xie Z. (2001). DAMBE: software package for data analysis in molecular biology and evolution. Journal of Heredity.

[B8142246] Yang Jin-Shu, Nagasawa Hiromichi, Fujiwara Yoshihiro, Tsuchida Shinji, Yang Wei-Jun (2008). The complete mitochondrial genome sequence of the hydrothermal vent galatheid crab *Shinkaiacrosnieri* (Crustacea: Decapoda: Anomura): a novel arrangement and incomplete tRNA suite.. BMC Genomics.

