# Supplementary material for: Novel gene re-arrangement in the mitochondrial genome of Pisidiaserratifrons (Anomura, Galatheoidea, Porcellanidae) and phylogenetic associations in Anomura
Source: Biodivers Data J. 2023 Feb 22;11:e96231. doi: 10.3897/BDJ.11.e96231 (PMC10848379; doi:10.3897/BDJ.11.e96231)
Supplement: Supplementary material 2 — List of 34 species and two outgroups used in this paper [file bdj-11-e96231-s002.doc]

Table 1 List of 34 species and two outgroup used in this paper.

| **Superfamily** | **Family** | **Species** | **Size** | **Accession.no** |
| --- | --- | --- | --- | --- |
| **Galatheoidea** | Porcellanidae | *Pisidia serratifrons* | 15,344 | OM461359 |
|  |  | *Neopetrolisthes maculatus* | 15,324 | KC107816 |
|  |  | *Petrolisthes haswelli* | 15,348 | NC_025572 |
|  | Munididae | *Munida gregaria* | 16,326 | NC_030255 |
|  |  | *Munida isos* | 17,910 | MF457406 |
|  | Munidopsidae | *Munidopsis Verrilli* | 17,636 | MH717896 |
|  |  | *Munidopsis lauensis* | 17,483 | MH717895 |
|  |  | *Shinkaia crosnieri* | 15,182 | EU420129 |
| **Paguroidea** | Diogenidae | *Dardanus arrosor* | 16,592 | MW147148 |
|  |  | *Dardanus aspersus* | 16,916 | MW715812 |
|  |  | *Clibanarius infraspinatus* | 16,504 | NC_025776 |
|  | Paguridae | *Pagurus similis* | 17,100 | NC_057304 |
|  |  | *Pagurus nigrofascia* | 15,423 | NC_042412 |
|  |  | *Pagurus gracilipes* | 16,051 | LC222534 |
|  |  | *Pagurus minutus* | 14,939 | LC222533 |
|  |  | *Pagurus japonicus* | 16,401 | LC222532 |
|  |  | *Pagurus longicarpus* | 15,630 | NC_003058 |
|  |  | *Pagurus lanuginosus* | 14,632 | LC222527 |
|  |  | *Pagurus maculosus* | 15,420 | LC222524 |
|  |  | *Pagurus sp.* | 14,648 | LC222535 |
|  |  | *Pagurus filholi* | 15,674 | LC222528 |
|  | Coenobitidae | *Coenobita rugosus* | 16,433 | MN030161 |
|  |  | *Coenobita brevimanus* | 16,388 | MN030160 |
|  |  | *Coenobita variabilis* | 16,421 | KY352236 |
|  |  | *Coenobita perlatus* | 16,447 | KY352234 |
|  |  | *Birgus latro* | 16,411 | NC_045091 |
|  | Lithodidae | *Paralithodes platypus* | 16,883 | NC_042240 |
|  |  | *Paralithodes camtschaticus* | 16,720 | NC_020029 |
|  |  | *Lithodes nintokuae* | 15,731 | NC_024202 |
| **Hippoidea** | Albuneidae | *Stemonopa insignis* | 15,596 | KY352240 |
| **Lomoidea** | Lomidae | *Lomis hirta* | 17,239 | KY352239 |
| **Chirostyloidea** | Kiwaidae | *Kiwa tyleri* | 16,865 | NC_034927 |
|  | Chirostylidae | *Gastroptychus investigatoris* | 16,423 | KY352237 |
|  |  | *Gastroptychus rogeri* | 16,504 | KY352238 |
|  | Outgroup | *Ocypode ceratophthalmus* | 15,555 | MW255974 |
|  |  | *Ocypode stimpsoni* | 15,557 | NC_046797 |
