# Supplementary material for: Novel gene re-arrangement in the mitochondrial genome of Pisidiaserratifrons (Anomura, Galatheoidea, Porcellanidae) and phylogenetic associations in Anomura
Source: Biodivers Data J. 2023 Feb 22;11:e96231. doi: 10.3897/BDJ.11.e96231 (PMC10848379; doi:10.3897/BDJ.11.e96231)
Supplement: Supplementary material 3 — Basic information on three fossil correction points [file bdj-11-e96231-s003.docx]

Table 2 Basic information on 3 fossil correction points.

| Taxonomy | Species | Geological Age (MYA) | Note |
| --- | --- | --- | --- |
| munididae | Juracrista perculta (Feldmann, and Schweitzer, 2012) | Late Jurassic (Tithonian) 145.5-151 | 1 |
| coenbitidae | Brigus latro(Linnaeus, 1767) | Pliocene 2.6-5.3 | 2 |
| munidopsidae | Palaeomunidopsis moutieri (Van Straelen, 1925) | Middle Jurassic (Bathonian) 168-165 | 3 |
